# Mutagenesis of *Puccinia graminis* f. sp. *tritici* and Selection of Gain-of-Virulence Mutants

**DOI:** 10.3389/fpls.2020.570180

**Published:** 2020-09-16

**Authors:** Ngonidzashe Kangara, Tomasz J. Kurowski, Guru V. Radhakrishnan, Sreya Ghosh, Nicola M. Cook, Guotai Yu, Sanu Arora, Brian J. Steffenson, Melania Figueroa, Fady Mohareb, Diane G. O. Saunders, Brande B. H. Wulff

**Affiliations:** ^1^Crop Genetics Department, John Innes Centre, Norwich, United Kingdom; ^2^The Bioinformatics Group, Cranfield Soil and Agrifood Institute, Cranfield University, Bedford, United Kingdom; ^3^Department of Plant Pathology, University of Minnesota, St. Paul, MN, United States; ^4^Agriculture and Food, Commonwealth Scientific and Industrial Research Organisation, Canberra, NSW, Australia

**Keywords:** *Puccinia graminis* f. sp. *tritici*, ethyl methanesulphonate mutagenesis, wheat, avirulence, effectors

## Abstract

Wheat stem rust caused by the fungus *Puccinia graminis* f. sp. *tritici* (*Pgt*), is regaining prominence due to the recent emergence of virulent isolates and epidemics in Africa, Europe and Central Asia. The development and deployment of wheat cultivars with multiple stem rust resistance (*Sr*) genes stacked together will provide durable resistance. However, certain disease resistance genes can suppress each other or fail in particular genetic backgrounds. Therefore, the function of each *Sr* gene must be confirmed after incorporation into an *Sr*-gene stack. This is difficult when using pathogen disease assays due to epistasis from recognition of multiple avirulence (Avr) effectors. Heterologous delivery of single *Avr* effectors can circumvent this limitation, but this strategy is currently limited by the paucity of cloned *Pgt Avrs*. To accelerate *Avr* gene cloning, we outline a procedure to develop a mutant population of *Pgt* spores and select for gain-of-virulence mutants. We used ethyl methanesulphonate (EMS) to mutagenize urediniospores and create a library of > 10,000 independent mutant isolates that were combined into 16 bulks of ~658 pustules each. We sequenced random mutants and determined the average mutation density to be 1 single nucleotide variant (SNV) per 258 kb. From this, we calculated that a minimum of three independently derived gain-of-virulence mutants is required to identify a given *Avr* gene. We inoculated the mutant library onto plants containing *Sr43*, *Sr44*, or *Sr45* and obtained 9, 4, and 14 mutants with virulence toward *Sr43*, *Sr44*, or *Sr45*, respectively. However, only mutants identified on *Sr43* and *Sr45* maintained their virulence when reinolculated onto the lines from which they were identified. We further characterized 8 mutants with virulence toward *Sr43*. These also maintained their virulence profile on the stem rust international differential set containing 20 *Sr* genes, indicating that they were most likely not accidental contaminants. In conclusion, our method allows selecting for virulent mutants toward targeted resistance (*R*) genes. The development of a mutant library from as little as 320 mg spores creates a resource that enables screening against several *R* genes without the need for multiple rounds of spore multiplication and mutagenesis.

## Introduction

Wheat stem rust is a destructive disease caused by the fungus *Puccinia graminis* f. sp. *tritici* (*Pgt*) that is resurging due to the evolution of virulent isolates that have overcome several stem rust resistance (*Sr*) genes (Pretorius et al., 2000; Olivera Firpo et al., 2017). Currently, 80% of the world’s wheat cultivars are vulnerable to infection (Singh et al., 2008; Lewis et al., 2018) and worldwide yearly grain losses attributed to the disease are estimated at 6.2 million tonnes, equivalent to ~1% of the annual wheat yield (valued at USD 1.12 billion; (Beddow et al., 2013). However, this masks crop losses at local or regional levels, which can reach 40% or more (Schumann and Leonard, 2000; Saunders et al., 2019).

Many plant pests and pathogens, including *Pgt*, deliver effector molecules into host cells to facilitate successful parasitism. These effectors serve to suppress host defenses by inhibiting immune response-signaling following pathogen invasion. Effectors can also regulate host gene expression (Ahmed et al., 2018) and play a role in nutrient acquisition during rust infection (Sohn et al., 2000; Thara et al., 2003). Some effectors can be detected by the products of host *R* genes encoding either extracellular or intracellular immune receptors, leading to effector triggered immunity (Dodds and Rathjen, 2010; Lo Presti et al., 2015). Such effectors are termed avirulence (Avr) effectors and are often associated with a macroscopic hypersensitive cell death response.

The deployment of a single *R* gene in a disease hotspot leads to a large selection pressure which typically results in the rapid emergence of resistance-breaking strains of the pathogen (Johnson, 1961; Vale et al., 2001; Hovmøller and Justesen, 2007). However, the judicious stacking of multiple *R* genes would, from first principle, maximize the durability of resistance as there would be no selective advantage to a pathogen isolate which has overcome just a single *R* gene in the stack (Huang et al., 2006; Zhang et al., 2009; Fukuoka et al., 2015). To functionally test *R* genes in such stacks on a one-to-one basis first requires the identification of their corresponding Avr effectors. Certain *R* genes can interfere with each other (Hurni et al., 2014), do not work in certain backgrounds (Hiebert et al., 2020), or risk being silenced when delivered as transgenes (Anand et al., 2003; Li et al., 2005). Thus, it is essential to test the individual function of each *R* gene in a stack. As pathogens deliver multiple effectors, disease resistance cannot always be used to test the function of every gene in the stack (Vleeshouwers and Oliver, 2014; Wulff and Moscou, 2014; Chen et al., 2017; Salcedo et al., 2017). Therefore, Avr effectors can be used as probes to confirm the function of *R* genes in the absence of the pathogen (Upadhyaya et al., 2014; Vleeshouwers and Oliver, 2014; Bouton et al., 2018; Saur et al., 2019). This property makes cloned *Avr* genes useful tools in both fundamental and applied research. For example, effectors have been used extensively to study *R* gene structure/function relationships (Wulff et al., 2009; Maqbool et al., 2015; Ortiz et al., 2017; Seto et al., 2017) and to assist in the engineering of *R* genes with novel specificities (Harris et al., 2013; Segretin et al., 2014; Kim et al., 2016; De La Concepcion et al., 2019).

To date, several major dominant *Sr* genes have been cloned. Examples include *Sr13* (Zhang et al., 2017), *Sr21* (Chen et al., 2018), *Sr22* (Steuernagel et al., 2016), *Sr33* (Periyannan et al., 2013), *Sr35* (Saintenac et al., 2013), *Sr45* (Steuernagel et al., 2016), *Sr46* (Arora et al., 2019), *Sr50* (Mago et al., 2015) *Sr60* (Chen et al., 2019), and *SrTA1662* (Arora et al., 2019), with many more underway. These provide an excellent foundation for engineering multi-*Sr* gene stacks. In contrast, the cloning of their corresponding *Avr* genes has lagged behind. Most wheat rust (*Puccinia*) *Avr* gene cloning strategies have used genome sequencing followed by bioinformatics screens to shortlist candidate genes based on presence of a signal peptide, absence of a transmembrane domain, being cysteine-rich and size (< 300 amino acids), followed by subsequent transient heterologous expression assays to test function (Saunders et al., 2012; Upadhyaya et al., 2015; Anderson et al., 2016). Such strategies have been successful for other pathogens such as *Blumeria graminis* (Pedersen et al., 2012; Ahmed et al., 2015) *Phytophthora* sp. (Vleeshouwers et al., 2008)*, Bremia lactucae* (Stassen et al., 2012), and *Magnaporthe oryzae* (Chen et al., 2013). However, for most rust fungi these approaches have been hampered by the lack of specific conserved sequence features for classifying effector genes and the absence of reproducible, genotype-independent and high-throughput transient assays for testing function. Moreover, conventional bi-parental genetics and positional cloning is impractical due to the difficulties in generating controlled sexual crosses and managing large numbers of segregating progeny (Johnson, 1954).

Mutagenesis followed by sequence-comparison of multiple independently-derived mutants presents an altogether different and promising approach, which has been successfully applied to identify genes from a wide array of organisms including worms, flies, and plants (Wang et al., 2010; Ashelford et al., 2011; Steuernagel et al., 2016; Addo-Quaye et al., 2017; Kawamura and Maruyama, 2019). *Pgt* work in the 1960s and 1970s showed that it is possible to mutagenize rust spores and select mutants that have lost the function of defined *Avr* genes (Teo and Baker, 1966; Luig, 1978). With the advent of genome sequencing, the first *Pgt Avr* effectors (*AvrSr35* and *AvrSr50*) were cloned by comparing induced or natural mutants to their wild type parents (Chen et al., 2017; Salcedo et al., 2017). The mutagenesis and screening methods described in these historical and recent studies, however, are lacking in detail and hence difficult to reproduce without significant investment in method optimization.

**Figure 1:**
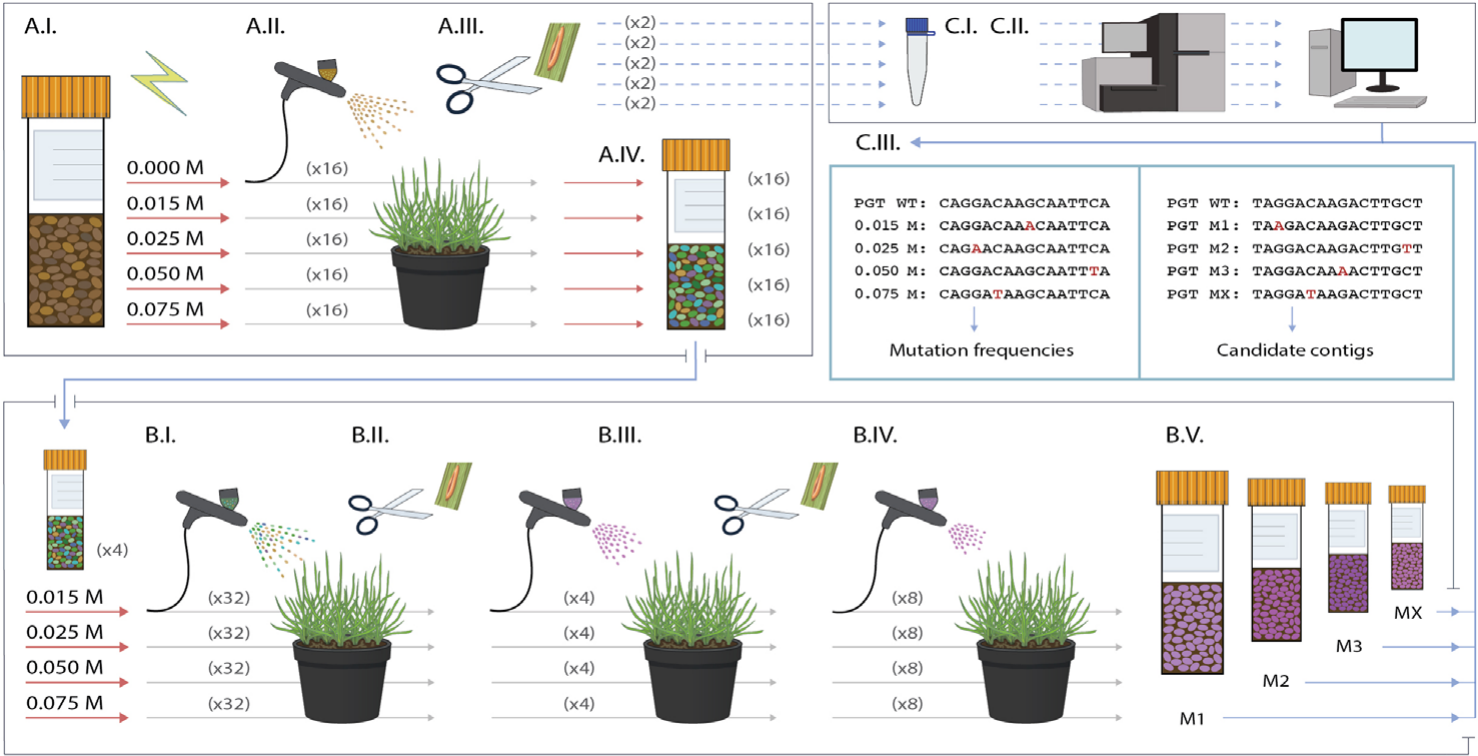
Overview of the development, screening and analysis of *Pgt* ethyl methanesulphonate (EMS) mutants. Development of a *Pgt* mutant library starting with mutagenesis of the wild type **(A.I)**, inoculation of 16 pots each containing seven susceptible wheat plants per EMS treatment **(A.II)**, selection of two single pustule samples per treatment for spore increase, DNA extraction and sequencing **(A.III)**, followed by spore harvesting from each separate pot resulting in 16 mutant spore bulks per treatment **(A.IV)**. The 16 bulks were pooled to make four master bulks per EMS treatment **(B.I)** and each bulk was inoculated on eight pots containing seven plants of the *Sr* line of interest along with the *Pgt* wild type control. Virulent pustules were isolated **(B.II)**, reinoculated onto the *Sr* line they were identified from **(B.III)**, and pure, single pustules were isolated for bulking up for downstream experiments **(B.IV)** and sequencing **(B.V)**. Sample pustules from each EMS treatment were sequenced to determine single nucleotide variant (SNV) rates, while virulent mutants can be sequenced and analyzed to identify *Avr* candidate genes **(C.III)**.

In the present study, we optimized procedures for generating mutant libraries of *Pgt* isolate UK-01, race TKTTF, using the chemical mutagen ethyl methanesulphonate (EMS) (**Figure 1**). We provide a detailed protocol on how to perform mutagenesis, identify the optimal concentration of EMS, select virulent mutants toward defined *Sr* genes, purify mutants, confirm their specificity and multiply purified spores for DNA extraction. We demonstrate this with screens against *Sr43*, *Sr44*, and *Sr45* in wheat. In order to accurately identify EMS-induced mutations, we generated a draft genome assembly of the wild type *Pgt* isolate UK-01 and conducted whole-genome resequencing of eight EMS-derived monopustule mutants to calculate mutation density. Using these data, we make theoretical predictions on the number of independent virulence mutants that would require resequencing to identify causative mutations.

## Materials and Methods

### Selection of *Aegilops tauschii* Lines Carrying Only *Sr45*, *Sr46*, or *SrTA1662*

The three genes, *Sr45*, *Sr46*, and *SrTA1662*, used to screen the mutant population were cloned from *Ae. tauschii* species (Steuernagel et al., 2016; Arora et al., 2019). To select the lines carrying only *Sr45*, *Sr46*, or *SrTA1662*, we inferred the presence of these genes by BLAST search (using a ≥ 99.8% identity and > 90% query coverage cut-off) in a panel of 151 *Ae. tauschii* accessions which had their NLR repertoires sequenced (Arora et al., 2019). In this way, we identified accessions carrying only one of the three genes – TOWWC0191 for *Sr45*, TOWWC0152 for *Sr46 *and TOWWC0017 for* SrTA1662*. These accessions were then screened with *Pgt* UK-01 and all lines were found to be resistant, indicating that *Pgt* UK-01 is avirulent toward *Sr45*, *Sr46*,* *and *SrTA1662*. These accessions are available from the Germplasm Resource Unit at the John Innes Centre, UK.

### Identifying Wheat-Alien *Sr* Introgression Lines Resistant to *Pgt* UK-01

Wild type *Pgt* UK-01 (race TKTTF) urediniospores were harvested 28 days after inoculation (see below) of cv. Vuka following single pustule isolation. The freshly harvested spores were placed in vials plugged with cotton wool and dried for four days using silica beads at room temperature and then stored at −80°C until use.

Seedlings of wheat or *Aegilops* lines containing the *Sr* genes *Sr22*, *Sr25*, *Sr33*, *Sr40*, *Sr43*, *Sr44*, *Sr45*, *Sr46*, *Sr51*, *Sr53*, *SrTA1662*, *Sr1644-1Sh*, *Sr2020*, recurrent parents, and the universal susceptible control wheat cvs Vuka and Chinese Spring (**Table 1**) were prepared by sowing two pots per line, each containing eight seeds. The seedlings were grown in a controlled environment room in a 23°C, 16 h light/15°C, 8 h dark cycle. Upon emergence of the coleoptile at approximately six days after germination the plants were treated with 0.2 g/L Maleic hydrazide solution (**Supplementary Table 1**) which was applied as a 50 ml pot drench. This stunted plant growth and enhanced leaf width.

**Table 1:** *Pgt* UK-01 infection types on *Sr* introgression lines and their recurrent parents, and *Aegilops* accessions predicted to carry single *Sr* genes.

*Sr* gene	*Sr* gene source	Infection type	Recurrent parent	Recurrent parent infection type	Selected for further analysis
*Sr22*	*Triticum monococcum*	1+2	Schomburgk	Not tested	Yes
*Sr25*	*Thinopyrum ponticum*	;1−	Louise	1−	No
*Sr33*	*Ae. tauschii*	22+	Chinese Spring	4	Yes
*Sr43*	*Th. ponticum*	1+2−	Chinese Spring	4	Yes
*Sr45*	*Ae. tauschii*	1−	Chinese Spring	4	Yes
*Sr46*	*Ae. tauschii*	1−	–	–	No
*Sr51*	*Ae. searsii*	1+2-	Chinese Spring	4	Yes
*Sr53*	*Ae. geniculata*	1	Chinese Spring	4	Yes
*Sr40*	*T. timopheevii* ssp. *armeniacum*	;1−	Westonia	4	Yes
*Sr44*	*Th. intermedium*	;	Angus	4	Yes
*Sr1644-1Sh*	*Ae. sharonensis*	1−	Zahir	;1−	No
*SrTA1662*	*Ae. tauschii*	1−	–	–	No
*Sr2020*	*Ae. sharonensis*	21	–	–	No
*Sr31* (resistant control)	*Secale cereale*	1−	Kavkaz	–	No
Vuka (susceptible control)		32+	–	–	No

For inoculations, urediniospores were taken from cold storage and “heat shocked” in a water bath at 45°C for 10 min. Each wheat or *Aegilops* line had three pots containing seven seedlings. The inoculum was applied to the leaves of the seedlings with an airbrush at a rate of 8 mg in 10 ml of 3 M™ Novec 7000™ Engineered Fluid (Novec) (**Supplementary Table 1**) for every eight pots (56 plants). Following inoculation, misting was conducted in the dark for 24 h at room temperature by placing the seedling pots in plastic bags and adding water to cover the bottom at a depth of about 5 cm. The open ends of the bags were sealed with cable ties. After 24 h, the pots were removed from the misting bags, re-bagged in breathable cellophane cross bottom bags fastened to the pots by elastic bands, and then transferred to the growth room with the previously described conditions. Phenotyping was conducted at 14 dpi using the Stakman *Pgt* phenotyping scale (Stakman et al., 1962).

### Mutant Library Creation

We prepared sixteen pots of 12- to 14-day-old wheat seedlings of the universally susceptible cultivar Chinese Spring by sowing seven wheat seeds per 9 × 9 cm pot. The seedlings were grown in a controlled environment room as described above. For each EMS mutagenesis experiment, 200 mg of *Pgt* UK-01 urediniospores were “heat shocked” in a water bath (**Supplementary Table 1**) at 45°C for 10 min. Solutions of four EMS concentrations—0.015 M, 0.025 M, 0.05 M, 0.075 M plus a water control—were prepared in the fume hood with sterile H_2_O containing 0.01% Tween 20 in 50 ml Greiner tubes (**Supplementary Table 1**). We added 40 mg of urediniospores to each tube and gently shook the suspensions by hand every 20 min over 1 h and 20 min at room temperature. The spore suspensions were collected separately by gravity filtration through a Whatman cellulose filter paper (**Supplementary Table 1**). The spores on each filter paper were washed with 500 ml of water with 0.01% Tween 20. After the water had drained, the urediniospores were washed off the filter paper using sterile water with 0.01% Tween 20 into 30 ml Nalgene™ Oakridge tubes (**Supplementary Table 1**). All EMS contaminated apparatus was immersed in EMS inactivation solution (0.1 M NaOH + 10% w/v Na_2_S_2_O_3_) for at least 24 h in the fume hood.

We then proceeded to inoculate cv. Chinese Spring plants in a Class 2 biological safety cabinet using the mutagenized and control urediniospores suspended in water with 0.01% Tween 20. All 30 ml of the inoculum were used for each set of 16 pots containing a total of 112 plants (**Figure 1**). The procedures for misting and onward growth of the plants was as described above. At 12 dpi, three single pustules per treatment were randomly selected for bulking up and sequencing to determine mutation frequencies. After this, the cellophane bags (**Supplementary Table 1**) were bent sideways to allow the spores to collect in the bags, except for four randomly selected pots per treatment which were kept aside for conducting pustule counts at 14 dpi. At 35 dpi, the spores were collected from the cellophane bags. Spores were dried and stored as described above.

### DNA Extraction and Whole-Genome Shotgun Sequencing

Sample pustules were harvested, diluted with Novec, and then inoculated onto four pots containing seedlings of cv. Vuka. The mono-pustule isolations were conducted twice. In the third cycle, 70–100 mg of urediniospores per sample were used for a CTAB DNA extraction. Urediniospores were mixed with 50 mg of glucose/sucrose mix and 50 mg sand and ground to a fine powder in a pestle and mortar. Prewarmed CTAB buffer (**Supplementary Table 1**) (50°C) was added to the ground mix followed by 10 µl proteinase K (20 mg/ml) and incubated at 50°C for 2 h with intermittent shaking. Another 10 µl proteinase K (20 mg/ml) was added followed by incubation at 50°C for 1 h. After this, 1 volume (V) chloroform:isoamyl alcohol (24:1) was added and the mix shaken vigorously and centrifuged at 5,000 g for 10 min. A total of 20 µl RNase (0.1 mg/ml) (**Supplementary Table 1**) was then added and incubated at room temperature for 1 h. A second chloroform:isoamyl extraction and centrifugation step was then conducted. DNA was precipitated from the aqueous phase using 1 V chilled (−20°C) isopropanol and left overnight in a −20°C freezer. DNA was pelleted by centrifugation at 16,000 g for 10 min. The DNA pellets were washed twice with 1 ml of 70% chilled ethanol and centrifuged at 16,000 g after each wash. The DNA pellets were dried and then suspended in 70 µl 1% TE buffer. DNA quantification and quality analysis was carried out using a Nanodrop™ spectrophotometer (ThermoFisher Scientific) and agarose gel electrophoresis and comparison to known concentrations of Lambda phage DNA. Illumina 350 bp insert library preparation and 150 bp paired-end whole genome shotgun sequencing was conducted at Novogene, Beijing or Genewiz.

### MinION Library Preparation, Sequencing and Assembly

High molecular weight genomic DNA was obtained from *Pgt* UK-01 using a protocol developed by Nagar and Schwessinger (2018). All steps were followed with the following minor changes: Firstly, the mixture of the lysis buffer and ground spores was left to stand for 30 min before adding Proteinase K. Secondly, after recovering the aqueous phase from the first chloroform:isoamyl alcohol (24:1) step, 0.1 V of 3 M sodium acetate (pH 5.2) was added before DNA was precipitated using 1 V isopropanol, followed by pelleting at 16,000 g for 5 min, followed by washing the DNA pellet with 70% ethanol, drying and elution in 500 µl 10 mM Tris HCl. Thirdly, RNase digestion for 1 h followed by Proteinase K digestion was conducted according to the protocol before the second (final) chloroform:isoamyl alcohol (24:1) (**Supplementary Table 1**) step followed by DNA precipitation using 1 V isopropanol, pelleting, ethanol washing, DNA pellet drying as previously described and elution in 200 µl TE buffer. The DNA was then prepared for sequencing on the MinION sequencer (Oxford Nanopore Technologies, Oxford, UK). The DNA concentration was measured using a Qubit fluorometer (Thermo Fisher Scientific). A total of 305 ng of DNA was used as input for library preparation using the 1D Ligation Sequencing Kit (SQK-LSK109, Oxford Nanopore Technologies) carried out as per the manufacturer’s instructions. The resulting library had a total mass of 67 ng and was sequenced on the MinION using a FLO-MIN106D flow cell (Oxford Nanopore Technologies) with 1,378 pores available for sequencing, following the manufacturer’s instructions, for a total of 48 h.

Basecalling of the MinION reads was performed using Guppy v3.0.3 (https://community.nanoporetech.com) on CPU mode using the default parameters. Following basecalling, only reads longer than 1 kb were taken forward for genome assembly using Canu v1.8 (Koren et al., 2017) using the default parameters and an estimated genome size of 170 Mbp. Genome completeness was assessed using BUSCO v3. (Waterhouse et al., 2018) for the Basidiomycota fungal lineage on genome mode with *Ustilago maydis* as the reference species for gene prediction using Augustus v3.2.1 (Stanke and Morgenstern, 2005).

### Polishing of the Nanopore Assembly

In order to polish the draft assembly, we generated PCR Illumina data by sequencing 250 bp paired-end Illumina reads with average insert size of 450 bp. We obtained three data sets containing a total of 19.7 Gbp which we aligned to the contigs of the Nanopore assembly using BWA-MEM 0.7.15 (Li and Durbin, 2009). The alignment results were sorted and duplicates marked using Picard 2.18 (Broad Institute, 2009) as the data were generated from PCR libraries. The alignment data were then used to improve the draft assembly using Pilon 1.23 (Walker et al., 2014) with the diploid setting and the default fix list: attempting to correct individual base errors, indel errors and local misassemblies, as well as fill gaps. This polishing workflow was repeated five times until the number of changes applied to the draft assembly plateaued. The resulting polished genome was re-assessed using BUSCO v3 (**Supplementary Table 2**) and used as the reference for downstream analysis.

### Determining EMS Mutation Rates

Whole genome resequencing data from eight EMS-derived mutants consisting of 150 bp paired-end Illumina reads were aligned to the polished *Pgt* UK-01 genome assembly using BWA-MEM 0.7.15. As with other Illumina alignments, the results were sorted and duplicates marked using Picard 2.18. The GATK 3.8 (McKenna et al., 2010) IndelRealigner tool was then used to create the final BAM alignment files for each of the mutants. Variant calling was then carried out using two different tools, GATK 3.8 HaplotypeCaller and bcftools 1.6 within Samtools (Danecek et al., 2011), yielding two sets of results per sample. The results were filtered with a basic GATK hard filter, removing low-quality or low-depth calls using the filtering parameters Quality of Depth (QD) < 2 and Mapping Quality (MQ) < 40. The entire pipeline was carried out for each of the eight mutant samples separately.

Seven of the eight samples (B1, B2, C2, D1, D2, E1, E3) in both sets of variant calls were then passed through a filtering pipeline aimed at identifying EMS-induced SNVs. The eighth sample (A1) was the control not treated with EMS, therefore any variants called for it were assumed to represent heterozygous variants already present in the genome prior to applying the mutagen, and thus most likely shared with the other samples. Tersect 0.12 (Kurowski and Mohareb, 2020) was used to remove A1 variants from the other seven variant sets. Variants appearing in three or more of the samples were also removed, as identical mutations are unlikely to have been induced independently. Finally, the variants were filtered to remove all except SNVs known to be preferentially induced by the EMS mutagen, that is G to A and C to T transitions.

To eliminate likely false positives the two sets of variant calling results were then intersected, retaining only SNVs called by both GATK HaplotypeCaller and bcftools to create “high confidence” sets of SNVs for each mutant. These high confidence sets were then used to estimate the EMS mutation rate related to different concentrations of EMS used for each sample.

### Calculating the Minimum Number of Independently Derived *Pgt* Mutants to Confidently Identify Candidate *Avrs*

For an unannotated genome to be used for comparative mutational genomics, every contig has to be treated as if it contains a potential gene candidate. If all the contigs in a given genome assembly are of length *l*, have the same GC content, and the canonical EMS mutations are distributed randomly across the assembly with a mutation density *m*, then, following the principles of binomial distribution, the probability (*P*) of a contig in such a genome assembly having mutations in *n* number of mutants by chance alone is:

$$P={\left(lm\right)}^{n}$$

where, *l* = length of the gene (number of bases)

*m* = mutation density (number of SNVs per base of the genome)

*n* = number of mutants

If the number of contigs of length *l* in such a genome assembly is *G*, then the number of contigs that are likely to have mutations in all *n* mutants by chance alone, i.e., the number of false positive candidates, *F*, is:

F=G×P

where, *G* = number of contigs in the assembly

*P* = probability of a contig in the assembly having mutations in all the surveyed mutants by chance alone.

Or, *F* = *G* × (*lm*)*^n^*

Given that the contigs of the UK-01 assembly have a uniform GC-content (**Supplementary Figure S1**), and assuming that the EMS SNPs are distributed evenly (Farrell et al., 2014; Shirasawa et al., 2016) the probability of a false positive candidate is therefore a function of the length of the contig.

Analyses of previously sequenced stem rust genomes have estimated that every 10 kb of the genome contains ~2 genes (Duplessis, 2011; Li et al., 2019). Thus, contigs of 10 kb or more can potentially complicate the search for candidate genes with mutations in all the mutants, as some of the mutants could have mutations in one gene within the contig while the other mutants could have mutations in the other gene. Therefore, to make our calculations, we chopped the contigs that were larger than 5 kb into smaller 5 kb contigs. The resultant chopped assembly contained contigs ranging from 1.001 - 5.999 kb. Since the equation for false positive number calculations only holds for contig lengths of roughly equal sizes, the UK-01 assembly contigs were divided according to their sizes into bins of 100 bp range, i.e., 1,000–1,100 bp, 1,100–1,200 bp, 1,200–1,300 bp and so on. The number of false positives within each bin was calculated using the formula for determining the value of *F*, where *l* = average length of contig in that bin, and *G* = number of contigs in that bin, and *n* = 1. In this way, the number of false positives was calculated for each bin and then these values were summed up to give the overall number of false positives from the whole assembly. If the number of false positives equalled a number greater than or equal to 1, then the value of *n* would be increased by 1 and the exercise repeated until the value of *F* was less than 1. The value of *n* for which F equals less than 1 is thus considered as the minimum number of mutants required to identify a candidate gene through comparison of re-sequenced mutant genomes. The calculation of the GC content of the contigs, division of the assembly into contig-length bins and iterative calculations of false positives were performed using custom code.

### Screening for Gain-of-Virulence on *Sr44*, *Sr43*, and *Sr45*

We conducted the screen for gain-of-virulence pustules sequentially on each *Sr* line and per mutant batch and bulk. For each treatment, the sixteen vials of independent mutants were pooled into four bulks (a total of 16 bulks for all mutants across treatments) (**Figure 1**). Seedlings of introgression lines were prepared as eight pots per mutant bulk using the same Maleic hydrazide treatment as described above. For inoculations, 8 mg of spores for each *Pgt* bulk and the wild type control were weighed and then heat-shocked. Following this, the spores were suspended in Novec at a rate of 8 mg of spores in 10 ml Novec per eight pots (56 plants) using an airbrush. Misting, bagging and plant growth conditions were as described above. Gain-of-virulence pustules were recovered at 16 dpi, dried for four days using silica beads and stored at −80°C and then used to inoculate the lines on which they were identified. Pustules that maintained their phenotype were then isolated and bulked up on the lines they were identified on for further experiments.

### Race Typing of *Sr43* Gain-of-Virulence Mutants

Virulent pustules were purified, multiplied and inoculation of the stem rust differential set was conducted as described above in the *Pgt* mutant screen for virulent candidates. Race phenotyping was carried out at 16 dpi.

## Results

### Identification of *Sr* Genes Which Provide Effective Resistance to the *Pgt* Isolate UK-01

**Figure 2:**
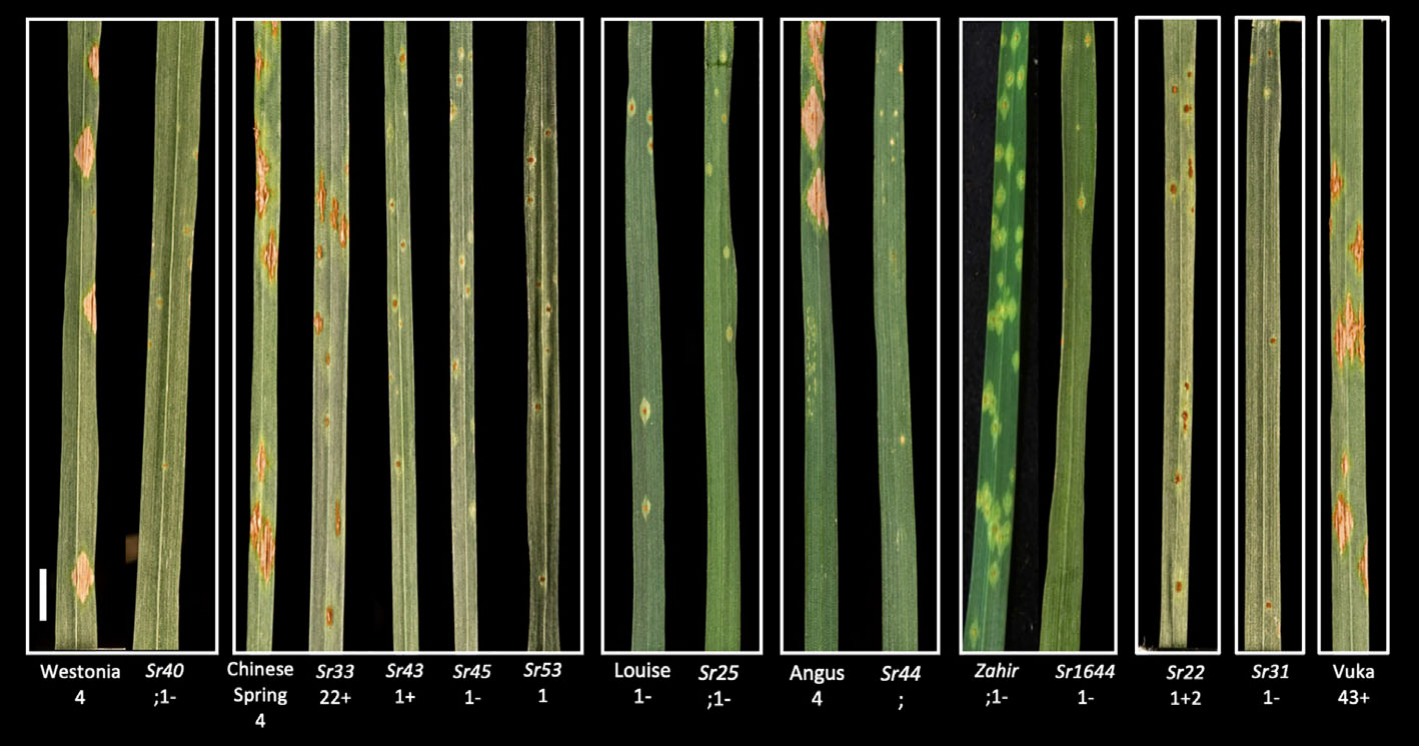
Infection types (Stakman et al., 1962) of *Pgt* UK-01, race TKTTF against nine *Sr* genes. *Pgt* UK-01 infection types scored at 14 dpi on a panel of wheat recurrent parents and their wheat-alien introgression progeny carrying single defined *Sr* genes, plus resistant *Sr31* (cv. Kavkaz/Federation4) and susceptible (cv. Vuka) control lines. *Sr40* exhibited strong resistance as opposed to its recurrent parent Westonia, which was susceptible. *Sr33* exhibited moderate resistance, while *Sr43*, *Sr45*, and *Sr53* displayed strong resistance as opposed to their recurrent parent Chinese Spring, which was susceptible. Although *Sr25* showed strong resistance, its recurrent parent Louise also exhibited strong resistance. *Sr44* and its recurrent parent Angus displayed distinct resistant and susceptible ITs. Both *Sr1644-1Sh* and its recurrent parent Zahir were resistant. *Sr22* was resistant while its recurrent parent Schomburgk (not shown) was susceptible. *Pgt* UK-01 was avirulent on the resistant control *Sr31*. Introgression lines are grouped in the same box with their recurrent parents except for *Sr22* and *Sr31*. The scale bar represents 1 cm.

To select *Pgt* mutants with induced mutations in a defined *Avr* gene requires a wheat line in which the corresponding *Sr* gene has been genetically isolated in a background which is susceptible to the *Pgt* isolate chosen for mutagenesis. To identify such *Sr* gene stocks for the *Pgt* isolate UK-01 which was designated race type TKTTF according to the North American nomenclature (Lewis et al., 2018), we inoculated: (i) ten wheat lines with chromosome segments carrying defined *Sr* genes from wild wheats (wheat-alien introgression lines), (ii) the wheat recurrent parents used for *Sr* introgression, (iii) two *Ae. tauschii* accessions predicted to carry only *Sr46* or *SrTA1662* (Arora et al., 2019), one *Ae*. *sharonensis* accession carrying *Sr2020*, and (iv) the respective susceptible and resistant control wheat cultivars Vuka and Kavkaz/Federation4 (*Sr31*) (**Table 1**, **Supplementary Table 3**). The comparison of infection types between an introgression line and its recurrent parent provided a basis for selection of the *Sr* genes most effective against UK-01 and a reference for the expected infection type of gain-of-virulence *Pgt* mutants (i.e., an infection type close to or equal to that of the recurrent parent). The phenotypes were assessed using the 0 to 4 scoring scale (Stakman et al., 1962). Scores from 0 to 2+ were considered avirulent, while scores from 3 to 4 were considered virulent. In total, ten *Sr* introgression lines and three *Aegilops* accessions exhibited a resistance response of 2+ or less (**Figure 2**). The resistance conferred by *Sr44* fully suppressed pustule development resulting in a fleck (;) infection type (IT) whereas its recurrent parent cv. Angus had an IT of 4. The lines *Sr22* (IT 1+2), *Sr33* (IT 22+), *Sr40* (IT ;1–), *Sr43* (IT 1+), *Sr44* (IT ;), *Sr45* (IT 1–), *Sr53* (IT 1), *Sr2020*, and *SrTA1662* (IT 1–), were considered suitable for selecting virulent mutant pustules because of their low IT compared to lines not carrying the respective *Sr* gene (**Table 1**). In contrast, *Sr1644-1Sh* (IT 1–) and *Sr25* (IT ;1) were not shortlisted as their recurrent parents Zahir and Louise, respectively, both exhibited ITs of ;1– and 1– to *Pgt* isolate UK-01 (**Figure 2**). In conclusion, we identified eleven *Sr* gene lines that exhibited sufficient resistance against the wild type *Pgt* isolate utilized to allow detection of gain-of-virulence mutant *Pgt* pustules.

### Development of the Mutagenesis Procedure

**Figure 3:**
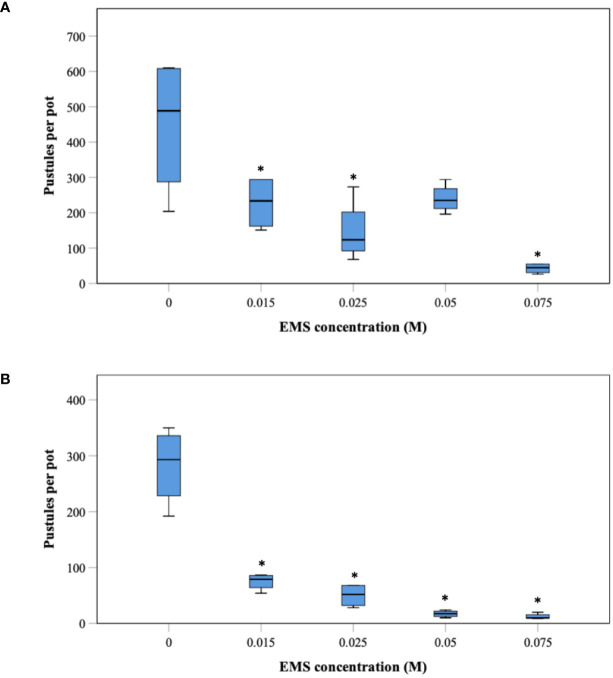
Pustule survival as an effect of different concentrations of the chemical mutagen ethyl methanesulphonate. Spores of *Pgt* UK-01 were subjected to different concentrations of ethyl methanesulphonate (EMS) and inoculated onto leaves of 12-day-old wheat seedlings of the susceptible cultivar Chinese Spring. For each EMS treatment, 4 of 16 pots were picked at random. Each pot contained seven plants. Pustules were counted at 14 dpi. The results from two independent experiments are displayed in [**A**, (EMS experiment 1)] and [**B**, (EMS experiment 2)]. Asterisks indicate significant differences between treatments and null control according to Fishers LSD test (**A**, P=0.001; **B**, P=0.000).

We conducted two *Pgt* mutagenesis experiments by incubating urediniospores in 0 M, 0.015 M, 0.025 M, 0.05 M, and 0.075 M EMS and inoculating the mutagenized spores onto 12 to 14-day old seedlings of the susceptible host cv. Chinese Spring (**Figure 1A, I–IV**). To determine the effect of EMS mutagenesis on spore viability, we counted the number of pustules appearing on seven seedlings from each EMS treatment as well as the water control at 13 days post inoculation (dpi) (**Supplementary Tables 4** and **5**). Analysis of pustule count as a function of EMS concentration revealed a clear decline in pustule survival as the EMS concentration increased (**Figure 3**).

Five weeks after inoculation, the resultant spores were harvested separately for each pot of seven seedlings. In each of the two EMS experiments, 16 pots were harvested per EMS concentration making a total of 128 mutant harvests (2 EMS experiments × 4 EMS concentrations × 16 pots = 128 harvests). The 16 mutant collections from each EMS concentration were then combined into four bulks to give a final set of 32 bulks—16 from each experiment. Based on the pustule counts at 14 dpi, we estimate that we obtained a total count of 10,520 and 2,496 mutant pustules in mutagenesis experiments 1 and 2, respectively (**Supplementary Tables 4** and **5**). Following this, the mutant population consisting of ca. 13,016 pustules was screened against selected *Sr* genes to identify candidate *Avr* genes.

### Sequencing and Assembly of the Wild Type UK-01 Reference Genome

We performed long-read sequencing of genomic DNA extracted from urediniospores of the wild type *Pgt* UK-01 isolate using the Oxford Nanopore MinION platform. We obtained 5.22 Gb of raw data, equivalent to an estimated 30-fold coverage of the ~170 Mbp dikaryotic *Pgt* genome (Li et al., 2019). The average read length was 2.58 kb, while the minimum and maximum read lengths were 0.08 and 51 kb, respectively (**Supplementary Table 2**). Only reads of a length greater than 1 kb were taken forward for assembly. We also generated 19.7 Gb of Illumina short read data from one 450 bp insert library with 250 bp paired-end reads. We first assembled the MinION reads with Canu and obtained an assembly size of 163.4 Mbp with 4,902 contigs and an N50 of 53.3 kbp (**Supplementary Table 2**). Assessment of genome completeness revealed that the Nanopore-only assembly contained over 93% of conserved fungal BUSCO genes (**Supplementary Table 2**). We then improved the assembly by incorporating the Illumina reads. Following polishing, the final assembly size was 164.3 Mbp. We therefore estimate that we assembled 96.6% of the ~170 Mbp dikaryotic *Pgt* genome.

### EMS-Induced Mutation Frequency in the *Pgt* Genome

To determine the genome-wide mutation density, we collected eight random pustules from the first EMS population (one to two pustules per EMS treatment) for mono-pustule isolation (**Figure 1A, III**) and sequencing (**Figure 1C, I–III**),. We aligned the raw reads for each of the mutants to the final wild type genome assembly and called single nucleotide variants (SNVs). To reduce the false discovery rate, we filtered the SNVs to (i) remove those SNVs which occurred in the nonmutated control, (ii) remove those SNVs that occurred in three or more mutants, and (iii) keep only the G:C to A:T transition mutations which typically account for around 98% of EMS-induced mutations (Lebkowski et al., 1986; Krasileva et al., 2017). The number of SNVs called by GATK (McKenna et al., 2010) per mutant genome ranged from 1,490 to 2,289, reflecting a G/C to A/T transition density per treatment of 1 per 103 kb for 0.015 M, 1 per 97 kb for 0.025 M, 1 per 87 kb for 0.05 M, and 1 per 80 kb for 0.075 M, which was similar in number to those called by SAMtools (Li et al., 2009) (**Table 2**). We then looked for overlap in the SNVs determined by GATK and Samtools to identify high confidence SNVs. This provided a high confidence SNV density of 1 per 404 kb for 0.015 M, 1 per 393 kb for 0.025 M, 1 per 239 kb for 0.05 M, and 1 per 182 kb for 0.075 M. The SNV densities were positively correlated with the EMS concentration (r^2^ = 0.9, in the case of the high confidence SNV densities) (**Supplementary Table 6**). The density of total, nonreduntant SNVs per treatment (i.e., high and low confidence SNVs identified by either program) ranged from 1 SNV per 56 kb to 1 SNV per 44 kb.

**Table 2:** Mutation frequency per ethyl methanesulphonate (EMS) treatment.

Sample	EMS level (M)	SNV count				Minimum no. of mutants required to identify causal mutations	
		GATK	Samtools mpileup	High confidence SNV	High + low confidence	Based on high confidence SNVs	Based on low confidence SNVs
				GATK + Samtools shared	Nonredundant SNVs		
B1	0.015	1,490	1,893	351	3,032	3	5
B2	0.015	1,678	2,358	524	3,512	3	5
C2	0.025	1,680	1,920	433	3,167	3	5
D1	0.05	1,861	2,307	701	3,467	3	5
D2	0.05	1,894	1,889	719	3,064	3	5
E1	0.075	2,289	2,599	1,033	3,855	4	5
E3	0.075	1,829	1,939	855	2,913	3	5

We calculated the minimum number of independently derived gain-of-virulence mutants required for sequence comparison with each other in order to identify the causative mutations and clone an *Avr* gene with confidence. We considered (i) a 5 kb window for contigs larger than 5 kb given that there is on average 1 gene per 5 kb in *Pgt* (Duplessis, 2011; Li et al., 2019), (ii) an observed mean GC content of 42.9% (with a standard deviation of 3.8%) per contig/window in *Pgt* UK-01 (**Supplementary Figure S1**), and (iii) the density of the SNV overlap determined by GATK and Samtools (**Table 2**). The total number of false positives (i.e., 1–5 kb windows with mutations in all compared mutants by chance alone) was then calculated for analysis using from 1 up to 10 gain-of-virulence mutants according to the principle of a binomial distribution. As a result, we determined the minimum number of independently derived *Pgt* mutants required to reduce the probability of calling false positives to zero was three across all EMS treatments (**Table 2**). This number was five when identifying causal gene *Avr* mutations using low confidence SNVs (**Table 2**). The higher number based on the total SNVs across both GATK and Samtools (i.e., nonreduntant SNVs) sets the minimum target for identification of gain-of-virulence mutants during *Pgt* mutant screens.

### EMS Mutagenesis of *Pgt* UK-01 Allows Recovery of Mutants With Stable Virulence to *Sr43* and *Sr45*, but Not *Sr44*

**Figure 4:**
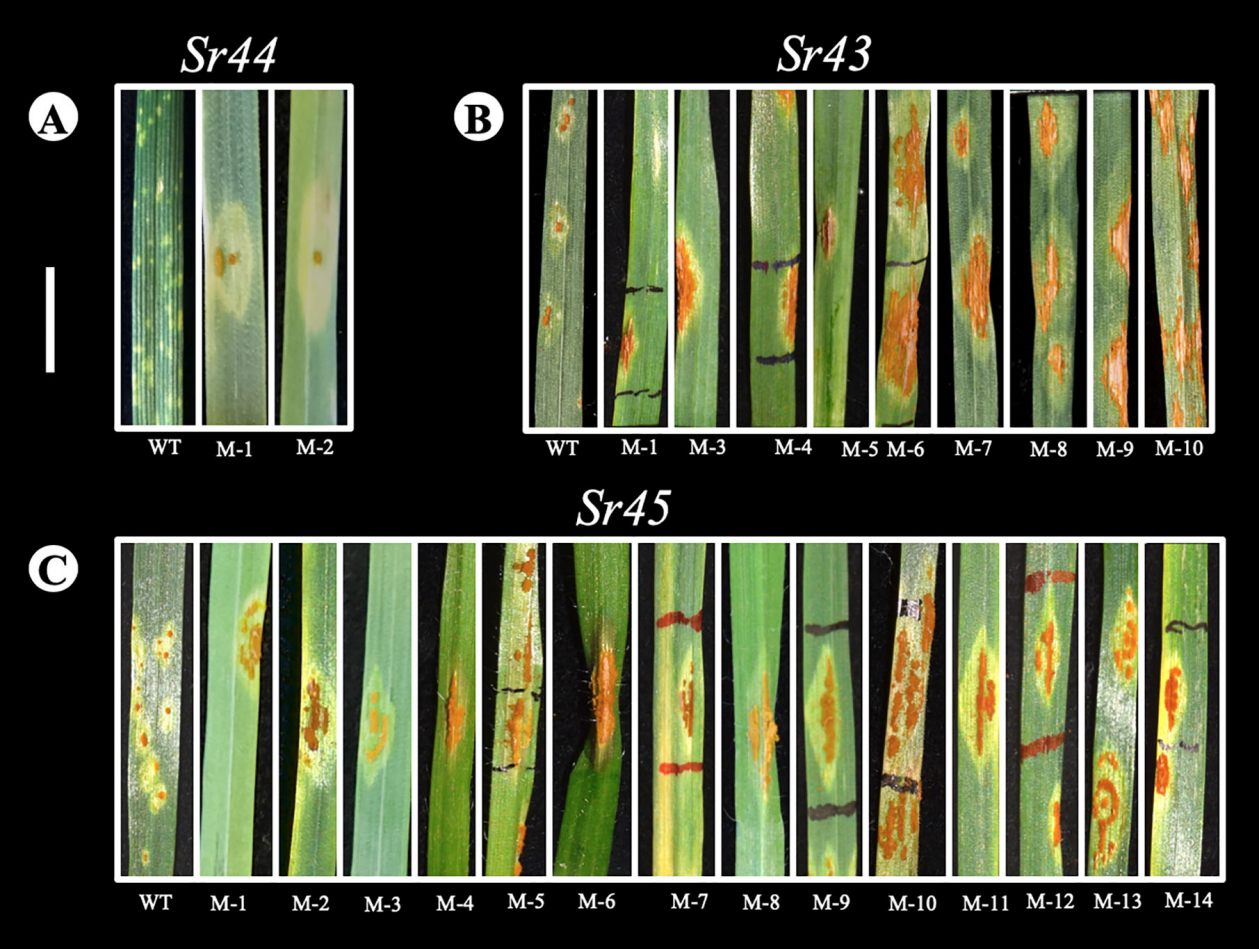
*Pgt* UK-01 EMS mutants with virulence to *Sr44* **[A]**, *Sr43* **[B]** and *Sr45* **[C]**. Virulent *Pgt* UK-01 mutant pustules obtained on Sr44, Sr43, and Sr45 were visually assessed at 16 dpi. Each mutant within a series was obtained from different mutant bulks and they were only selected if the pustule size was significantly larger than that of the wild type Pgt UK-01 control. Pustule infection type on the controls indicate resistance, *Sr44* IT ; **[A]**, *Sr43* IT +1 **[B]** and *Sr45* 1- **[C]**. Scale bar represents 1 cm.

We screened the *Pgt* mutant population from Experiment 1 comprising 10,520 independent pustules on the wheat-*Th. intermedium Sr44* introgression line. We identified four weakly virulent mutant pustules which displayed an IT of 1+ at 21 dpi (**Figure 4A**, mutants M-1 and M-2; **Supplementary Table 7**). However, the IT of these mutants reverted to wild type (;) after multiplication and re-inoculation onto *Sr44*. We then proceeded to screen both mutant libraries, i.e., 13,016 *Pgt* mutants, on the wheat-*Th. ponticum Sr43* line. Here we obtained nine gain-of-virulence mutants at 16 dpi (**Figure 4B**). Each virulent mutant had an IT of at least 3 or 4 while the IT of the wild type *Pgt* isolate UK-01 on *Sr43* was 1+ (**Figure 4B**, **Supplementary Table 7**). The mutant ITs became distinguishable from that of the wild type at 12 dpi with the pustules reaching full size at 16 dpi. Finally, we screened the 13,016 mutant pustules against *Sr45* (either the wheat-*Ae. tauschii Sr45* stock, or the *Ae. tauschii* lines predicted to only contain *Sr45*) and identified fourteen virulent mutants with ITs ranging from 2 to 4 (**Figure 4C**, **Supplementary Table 7**). The *avrSr43* and *avrSr45* mutants maintained their virulence after multiplication and re-inoculation onto the *Sr43* and *Sr45* stocks, respectively. When inoculated onto the cv. Chinese Spring recurrent parent the virulent mutants did not show any change in pustule color or morphology compared to that of the wild type, *Pgt* isolate UK-01. However, some of the mutants grew more slowly and produced fewer spores than the wild type likely due to background mutations affecting fitness.

### *Pgt* Mutants Virulent on *Sr43* Maintain the Same Virulence Profile as the Wild Type on the International Standard Differential Set

To test virulence specificity, we selected eight mutants with a gain-of-virulence on *Sr43* and inoculated them (alongside the *Pgt* UK-01 wild type control) onto the stem rust international differential set consisting of twenty lines with single defined *Sr* genes (Stakman et al., 1962). Infection types were observed between 14 and 16 dpi (**Supplementary Table 8**). Wild type *Pgt* UK-01 had low infection types on three (*Sr11*, *Sr24*, and *Sr31*) of the 20 lines, and the ITs of the nine mutants were indistinguishable from the wild type. Thus the mutants had the same race designation of TKTTF as defined by their virulence profile on the differential set. The mutants showed no additional virulence compared to the wild type on the 20 lines, apart from differing in *Sr43*.

## Discussion

The rapid advance of next-generation sequencing and computational technologies has enabled the cloning of several major dominant *Sr* genes and many more are in the process of being cloned (Keller et al., 2018; Periyannan, 2018). This has made the engineering of multi-*Sr* gene stacks for durable stem rust resistance a possibility. In contrast, the cloning of corresponding *Pgt Avr* genes has lagged behind as they do not have defined sequence signatures (unlike the *R* genes) making sequence analysis to identify *Avrs* difficult. To circumvent this and accelerate *Pgt Avr* gene cloning, we have outlined a procedure to generate mutant populations of *Pgt* spores and select for multiple gain-of-virulence mutants toward targeted *Sr* genes. Our work builds on historical studies from the **‘**60s and **‘**70s where chemical mutagens were used to obtain rust isolates virulent against *R* genes in oat and wheat (Teo and Baker, 1966; Luig, 1978). We innovated the development of bulks of mutant *Pgt* spores, creating a resource which enables screening against several *R* genes without the need for multiple rounds of spore bulking and mutagenesis.

### Identification of Effective *Sr* Gene Lines to Clone Corresponding *Avr* Genes

We tested 13 *Sr* genes for their efficacy in controlling wild type *Pgt* UK-01 in order to identify *Sr* gene targets suitable for *Pgt* gain-of-virulence screens. The ITs of the *Pgt* UK-01 isolate on the 13 lines predicted to carry single *Sr* genes of interest that met the cut-off criteria ranged from necrotic fleck (;) to intermediate resistance (2+) (Stakman et al., 1962). However, two of these 13 *Sr* genes, *Sr1644-1Sh* and *Sr25*, were not shortlisted because their recurrent parents Zahir and Louise had an IT of 1+2– indicating the presence of background resistance. Additional resistance in the recurrent parents would significantly reduce the probability of obtaining gain-of-virulence *Pgt* EMS mutants because it would require the rare simultaneous loss of more than one *Avr*.

Some of the shortlisted introgression lines carry *Sr* genes which have been previously cloned, including *Sr22*, *Sr45* (Steuernagel et al., 2016), *Sr33* (Periyannan et al., 2013), and *Sr46* (Arora et al., 2019). These present an opportunity for faster functional validation of the genetic interaction of corresponding *Sr-Avr* pairs by transient coexpression in wheat protoplasts (Saur et al., 2019), by virus-induced overexpression in leaves of seedlings (Bouton et al., 2018) or in a heterologous system like *Nicotiana benthamiana* (Salcedo et al., 2017). Furthermore, with the exception of *Sr22*, these *Sr* genes have not yet been deployed and provide resistance to races that are predominant in areas where *Pgt* is prevalent. Examples include: *Sr33* (Periyannan et al., 2013), *Sr43* (Yu et al., 2017) and *Sr45* (Steuernagel et al., 2016). In summary, 11 *Sr* gene lines which displayed a stronger resistance than their respective recurrent parent background were identified as suitable for selecting gain-of-virulence mutants.

### Creation of *Pgt* Mutant Libraries for Screening on Target *Sr* Introgression Lines

We present a robust and detailed step-by-step method for creating mutant *Pgt* libraries which can be returned to again and again as a resource for multiple mutant screens. We introduced a step where we inoculated mutagenized spores onto a susceptible cultivar, Chinese Spring, for propagating large amounts of mutant spores. Bagging of individual pots containing inoculated Chinese Spring plants prevented cross contamination and the spores from each pot were harvested separately. Creating a population of *Pgt* mutants allowed us to screen multiple *Sr* lines sequentially in a confined containment space, reduced the need for multiple rounds of mutagenesis and made extended use of the limited starting material of 400 mg of wild type urediniospores (including the control) in both experiments.

In previous *Pgt* EMS mutagenesis studies, freshly mutagenized spores were directly inoculated onto resistant plants carrying defined *Sr* genes (Teo and Baker, 1966; Luig, 1978; Salcedo et al., 2017). This requires a large amount of initial inoculum and generation of a new mutant library at the start of any new screen. In contrast, a mutant urediniospore library can be aliquoted and stored for long periods of time in a −80°C freezer or liquid nitrogen. We harvested mutant spores separately from each of the 16 pots per EMS treatment and pooled them into batches of four per EMS treatment (to create 16 bulks per experiment). This strategy allowed us to select independently derived gain-of-virulence mutants on either *Sr43* or *Sr45* as only one virulent pustule was selected per mutant bulk. This is an important consideration as multiple independent mutants are required to identify causative mutations through identification of a gene which is mutated in all or most individuals (Sánchez-Martín et al., 2016).

During mutant library creation, we observed a decline in pustule numbers with increasing EMS concentration (**Figure 3**) concomitant with a slight increase in high confidence SNV density from 1 SNV per 484 kb at the lowest EMS concentration to 1 SNV per 165 kb at the highest concentration (**Table 2**). This suggests a potential increase in deleterious mutations with increasing G/C to A/T mutation densities. The average mutation rate of 1 SNV per 258 kb for high confidence SNVs gave us the assurance that the mutagenesis had worked and screening for candidate loss of effector function mutants would be possible. In the previous *Pgt* mutagenesis screen which led to the cloning of the *AvrSr35* effector, the mutation density ranged from 1 SNV per 2,152 kb to 1 SNV per 10 kb with the average mutation rate being 1 SNV per 77 kb (Salcedo et al., 2017). Our mutation density for the nonredundant SNVs was slightly higher, but fell within the previously observed range between 1 SNV per 44 kb and 1 SNV per 54 kb. This 1.3-fold increase in the nonredundant SNV mutation density that we observed between the lowest and highest EMS concentration resulted in a 5 to 8-fold decrease in pustule number. However, there was no discernable difference in the frequency of obtaining gain-of-virulence mutants between the different EMS concentrations (expressed as number of mutants obtained per 10^6^ SNVs screened, **Supplementary Tables 4** and **5**). Therefore, the lower EMS concentrations used here are ideal for use in *Pgt* forward genetic screens because these concentrations maximize spore survival against only a minor decrease in mutation density.

We proceeded to screen the mutants on *Sr43* and *Sr45*. From these screens, we identified multiple gain- of-virulence mutants on introgression lines carrying *Sr43* (11 mutants) and *Sr45* (14 mutants). This suggests that the *Pgt* UK-01 isolate is likely heterozygous for the corresponding effectors. In contrast, we did not obtain any *bona fide* mutants for *Sr44*, suggesting that *Pgt* UK-01 may contain two functional copies of *AvrSr44* or that the introgressed *Th. intermedium* segment carries more than one *Sr* gene effective toward *Pgt* UK-01. Based on the average mutation rates within the *Pgt* genome, we calculated that at least three independently derived mutants would be required to clone a candidate *Avr* gene when considering high confidence SNVs, while five mutants are the minimum number when considering the high and low confidence SNVs. This assumes a “gene-for-gene” *Avr-Sr* interaction (Flor, 1955) whereby the change in *Pgt* phenotype in all the mutant gain-of-virulence individuals identified per *Sr* line can be attributed to a mutation in the corresponding *Avr* (rather than a second site suppressor of an *Avr* gene function) (Melania et al., 2020). The phenotypes of the gain-of-virulence mutants had no distinct differences from wild type *Pgt* UK-01 on Chinese Spring, the recurrent parent for both the *Sr43* and *Sr45* introgression lines. Teo and Baker (1966) observed a small number of EMS mutants of *P. graminis* f. sp. *avenae* on resistant lines, which developed teliospores early, while (Luig, 1978) obtained some *Pgt* pustules that were yellow or orange. Such phenotypes were not reported in *Pgt* mutants by Salcedo et al. (2017), nor in the present study.

Race typing of the *Sr43* gain-of-virulence mutants on the stem rust international differential set containing lines carrying 20 single *Sr* genes confirmed that the mutants retained the same TKTTF race designation of the wild type. This indicated that the gain-of-virulence observed was likely due to loss of a single effector gene (*AvrSr43*) rather than contamination from other isolates – as *Pgt* UK-01 was the only isolate used in the area where our experiments were conducted and *Pgt* UK-01 is the only isolate present in the UK (Lewis et al., 2018) – or gross genetic aberrations taking out multiple *Avr* genes. In this study, the general conservation in virulence profiles between the *Pgt* wild type isolate and mutants also provides further support to these being genuine derived mutants rather than potential contaminants.

We observed (but did not quantify) that some virulent mutants did not sporulate well during multiplication of pure spores for additional experiments. This did not correlate with the EMS dosage used to generate the mutants. In the example of mutants identified on the line with *Sr43*, mutant M-7 was derived from a bulk developed using 0.075 M EMS and was more prolific in spore production than M-3, derived from 0.025 M EMS. Thus, M-3 required two or three rounds of multiplication to produce the equivalent amount of spores. This is likely due to second-site mutations in M-3 which reduced fitness.

In summary, we have developed a detailed protocol for obtaining *Pgt* gain-of-virulence mutants. We demonstrate this by obtaining gain-of-virulence mutants toward either *Sr43* or *Sr45*. This work, coupled with the ability to obtain and analyze whole genome sequence data of multiple virulent mutants (Salcedo et al., 2017) presents an opportunity to clone the *Avrs* corresponding to *Sr43* and *Sr45*. Beyond this work, this method can be applied to obtain mutants in other *Avr* genes of *Pgt* as well as for other fungal pathogens with haploid genomes that can be propagated using asexual spores. With time, we expect the near complete pan-genome complement of *Pgt Avrs* to be cloned. This will facilitate large-scale sequence-based monitoring of *Pgt* virulence in the field and predictive breeding, including the creation and deployment of efficient stacks to control this important pathogen.

## Data Availability Statement

The datasets presented in this study can be found in online repositories. The names of the repository/repositories and accession number(s) can be found below: https://www.ebi.ac.uk/ena, PRJEB38623.

## Author Contributions

NK and GY conducted the *Pgt* phenotyping on the *Sr* panel. NK reduced to practice the *Pgt* mutagenesis process originally developed by MF and BS, conducted the *Pgt* mutagenesis, mutant screens, data collection, analysis, DNA extractions and drafted the manuscript. NC prepared libraries and setup the sequencing of the *Pgt* DNA libraries on the MinION. GR conducted processing, analysis and assembly of the wildtype Nanopore sequencing data. TK and FM conducted polishing of the Nanopore assembly and the EMS mutation frequency analysis. SG calculated the minimum number of mutants required to clone a high confidence candidate *Avr*. SA analyzed *Ae. tauschii* accessions to identify lines carrying only *SrTA1662*, *Sr45* or *Sr46*. MF and BS developed the original mutagenesis procedure adapted in this study and reviewed the manuscript. BW conceived the study. BW and DS designed and supervised the study, and revised the manuscript with input from GR, SG, NC, SA, TK, MF, and BS. All authors contributed to the article and approved the submitted version.

## Funding

We gratefully acknowledge funding from the European Union’s Horizon 2020 research and innovation programme under the Marie Skłodowska-Curie grant agreement No 674964, the John Innes Centre Science For Africa Initiative (JIC-SFA) and the Biotechnology and Biological Sciences Research Council (BBSRC) cross-institute strategic programmes Designing Future Wheat (BB/P016855/1) and Plant Health (BB/P012574/1) through a Discovery Fellowship to GR (BB/S011005/1). SG was supported by a Monsanto’s Beachell-Borlaug International Scholars’ Program fellowship and 2Blades Foundation.

## Conflict of Interest

The authors declare that the research was conducted in the absence of any commercial or financial relationships that could be construed as a potential conflict of interest.
