# Supplementary figures and images for: Mutagenesis of Puccinia graminis f. sp. tritici and Selection of Gain-of-Virulence Mutants
Source: Front Plant Sci. 2020 Sep 16;11:570180. doi: 10.3389/fpls.2020.570180 (PMC7533539; doi:10.3389/fpls.2020.570180)

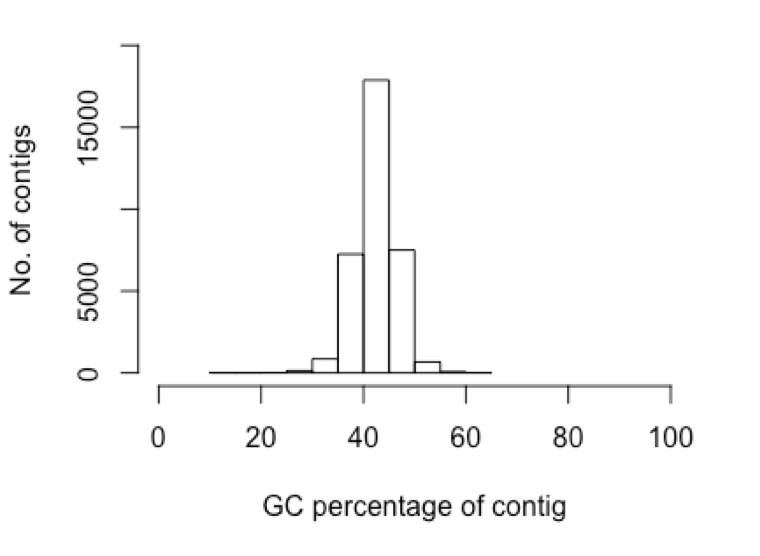

Supplement: Supplementary file 1 [file Image_1.png]
