# Supplementary material for: Mutagenesis of Puccinia graminis f. sp. tritici and Selection of Gain-of-Virulence Mutants
Source: Front Plant Sci. 2020 Sep 16;11:570180. doi: 10.3389/fpls.2020.570180 (PMC7533539; doi:10.3389/fpls.2020.570180)
Supplement: Supplementary file 2 [file Table_1.docx]

Supplementary Material

**Table S1 |** Materials that are required for the creation and screening of *Pgt* mutant libraries.

| Inoculations | *Pgt* wildtype or mutant urediniospores |
| --- | --- |
|  | Methanesulfonic acid ethyl ester (Sigma-Aldrich catalogue no. M0880) |
|  | EMS inactivation solution: 0.1 M NaOH + 10 % w/v Na_2_S_2_O_3_ |
|  | 30 ml Nalgene^TM^ Oakridge tubes (ThermoFisher Scientific catalogue no. 3119-0030PK) |
|  | 0.2 g/l Maleic hydrazide 0.2 g/l solution (Sigma-Aldrich catalogue no. D119806) |
|  | Tween20 |
|  | Grade 201 Whatman cellulose filter paper (GE healthcare Lifesciences, Europe) |
|  | Sterile H_2_O |
|  | 12 day-old Chinese Spring and *Sr* introgression line and seedlings in 9 cm pots |
|  | HS ENG^TM^ mini airbrush compressor AS18- 2^TM^ (Oil-free) |
|  | Siphon feed airbrush single action air brush kit 0.8 mm spray guns |
|  | Class 2 biological safety cabinet |
|  | 3M™ Novec™ 7000 Engineered Fluid (Sigma-Aldrich catalogue no. SHH0001) |
|  | Clear cellulose film cross bottom bags 180 mm x 300 (Helmut Schmidt Verpackungsfolien GmbH) |
|  | Biological waste bags |
|  | 70 % Ethanol |
|  | Elastic bands |
|  | Water bath set at 45 ^o^C |
|  | Glasshouse/ controlled environment growth room with temperature set at 21 – 23 ^o^C/ 15 ^o^C for 16 h/ 8 h temperature and 16 h fluorescent light. |
| Phenotyping, pustule sampling, spore harvesting and storage | Wheat stem rust phenotyping scale (Stakman et al. 1962) |
|  | Fine point scissors 12.5 cm |
|  | 2 ml centrifuge tubes |
|  | 1.5 ml Nalgene™ general long-term storage cryogenic tubes (ThermoFisher catalogue no. 5000-1020) |
|  | -80 ^o^C freezer |
|  | Fine point scissors 12.5 cm |
|  | Cotton wool |
| DNA extractions | Mortar and pestle |
|  | High molecular weight DNA extraction protocol (Nagar and Schwessinger, 2018) |
|  | 1 % agarose gel |
|  | Lambda DNA *Hind*III Digest (Sigma Aldrich catalogue no. D9780) |
|  | ThermoFisher Scientific NanoDrop™ 2000 spectrophotometer |
|  | Water bath set at 50 ^o^C |
|  | Proteinase K 20 mg/ml ThermoFisher Scientific (catalogue **no.**AM2548) |
|  | Rnase A 1 mg/ml ThermoFisher Scientific (catalogue **no.**AM2269) |
|  | CTAB |
|  | PVPP (insoluble P6755) |
|  | Glucose:sucrose, Merck (Catalogue **no.**s G8270 and S7903) |
|  | Sterile ddH_2_O |
|  | Chloroform:isoamyl alcohol (24:1) |
