# Supplementary material for: Mutagenesis of Puccinia graminis f. sp. tritici and Selection of Gain-of-Virulence Mutants
Source: Front Plant Sci. 2020 Sep 16;11:570180. doi: 10.3389/fpls.2020.570180 (PMC7533539; doi:10.3389/fpls.2020.570180)
Supplement: Supplementary file 3 [file Table_2.docx]

Supplementary Material

**
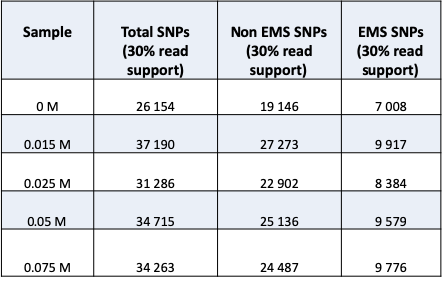
TABLE S2**⎟ *Pgt* UK-01 wildtype assembly statistics.

| **Parameters** | **Nanopore assembly** | **Polished assembly** |
| --- | --- | --- |
| No. of contigs | 4,902 | 4,902 |
| Contigs ≥ 10,000 bp | 3,956 | 3964 |
| Total length (Mb) | 163.4 | 164.3 |
| Total length ≥ 10,000 bp | 158.2 | 159.1 |
| N50 (kb) | 56.2 | 53.6 |
| Min (kb) | 1.01 | 1.01 |
| Max (kb) | 570.8 | 572.4 |
| L50 | 812 | 812 |
| GC (%) | 43.45 | 43.45 |
| % complete BUSCOs | 76.7 | 93.9 |
| % single-copy BUSCOs | 50.6 | 47.3 |
| % duplicated BUSCOs | 26.1 | 46.6 |
| % fragmented BUSCOs | 16.4 | 3.6 |
| % missing BUSCOs | 6.9 | 2.5 |
