# Supplementary material for: Mutagenesis of Puccinia graminis f. sp. tritici and Selection of Gain-of-Virulence Mutants
Source: Front Plant Sci. 2020 Sep 16;11:570180. doi: 10.3389/fpls.2020.570180 (PMC7533539; doi:10.3389/fpls.2020.570180)
Supplement: Supplementary file 5 [file Table_4.docx]

Supplementary Material

**TABLE S4**⎟ Number of *Pgt* pustules from creation of EMS population 1.

| **Pot number** | **Plant** | **0 M** | **0.015 M** | **0.025 M** | **0.05 M** | **0.075 M** |
| --- | --- | --- | --- | --- | --- | --- |
|  | 1 | 27 | 51 | 40 | 69 | 6 |
|  | 2 | 25 | 25 | 24 | 37 | 3 |
| Sample pot 1 | 3 | 49 | 60 | 40 | 19 | 10 |
|  | 4 | 37 | 33 | 52 | 26 | 7 |
|  | 5 | 15 | 40 | 24 | 52 | 7 |
|  | 6 | 31 | 42 | 29 | 61 | 11 |
|  | 7 | 20 | 43 | 35 | 30 | 11 |
|  | **Pot total** | **204** | **294** | **244** | **294** | **55** |
|  | 8 | 35 | 41 | 29 | 51 | 7 |
|  | 9 | 52 | 44 | 18 | 34 | 2 |
| Sample pot 2 | 10 | 105 | 49 | 19 | 94 | 5 |
|  | 11 | 56 | 60 | 10 | 49 | 6 |
|  | 12 | 55 | 38 | 25 | 42 | 4 |
|  | 13 | 84 | 62 | 44 | 13 | 8 |
|  | 14 | 64 | 37 | 15 | 9 | 3 |
|  | **Pot total** | **451** | **331** | **160** | **292** | **35** |
|  | 15 | 93 | 42 | 27 | 29 | 2 |
|  | 16 | 62 | 55 | 16 | 20 | 10 |
| Sample pot 3 | 17 | 62 | 39 | 25 | 22 | 7 |
|  | 18 | 82 | 30 | 40 | 28 | 0 |
|  | 19 | 40 | 33 | 16 | 33 | 2 |
|  | 20 | 37 | 30 | 19 | 40 | 0 |
|  | 21 | 69 | 33 | 14 | 35 | 3 |
|  | 22 | 81 | 25 | 27 | 31 | 4 |
|  | **Pot total** | **526** | **287** | **184** | **238** | **28** |
|  | 23 | 54 | 0 | 0 | 63 | 8 |
|  | 24 | 88 | 0 | 0 | 21 | 6 |
| Sample pot 4 | 25 | 79 | 0 | 0 | 29 | 10 |
|  | 26 | 79 | 0 | 0 | 23 | 6 |
|  | 27 | 38 | 0 | 0 | 0 | 8 |
|  | 28 | 100 | 0 | 0 | 0 | 12 |
|  | 29 | 88 | 0 | 0 | 0 | 4 |
|  | 30 | 84 | 0 | 0 | 0 | 0 |
|  | **Pot total** | **610** | **0** | **0** | **136** | **54** |
| **Total for 4 pots** | | **1791** | **912** | **588** | **960** | **172** |
| **Extrapolation to experiment total (16 pots)** | | **7164** | **3648** | **2352** | **3840** | **688** |
