# Supplementary material for: Mutagenesis of Puccinia graminis f. sp. tritici and Selection of Gain-of-Virulence Mutants
Source: Front Plant Sci. 2020 Sep 16;11:570180. doi: 10.3389/fpls.2020.570180 (PMC7533539; doi:10.3389/fpls.2020.570180)
Supplement: Supplementary file 6 [file Table_5.docx]

Supplementary Material

**TABLE S5**⎟ Number of *Pgt* pustules from creation of EMS population 2.

| **Pot number** | **Plant** | **0 M** | **0.015 M** | **0.025 M** | **0.05 M** | **0.075 M** |
| --- | --- | --- | --- | --- | --- | --- |
|  | 1 | 21 | 6 | 11 | 6 | 3 |
|  | 2 | 26 | 13 | 3 | 0 | 1 |
|  | 3 | 31 | 10 | 3 | 2 | 1 |
| Sample pot 1 | 4 | 14 | 10 | 6 | 7 | 2 |
|  | 5 | 23 | 11 | 3 | 2 | 0 |
|  | 6 | 29 | 20 | 4 | 0 | 1 |
|  | 7 | 21 | 17 | 1 | 1 | 0 |
|  | 8 | 27 | 0 | 5 | 2 | 1 |
|  | **Pot total** | **192** | **87** | **36** | **20** | **9** |
|  | 9 | 25 | 11 | 7 | 4 | 1 |
|  | 10 | 48 | 5 | 11 | 3 | 2 |
|  | 11 | 31 | 13 | 11 | 0 | 2 |
| Sample pot 2 | 12 | 42 | 5 | 14 | 0 | 8 |
|  | 13 | 40 | 8 | 5 | 1 | 3 |
|  | 14 | 49 | 14 | 10 | 4 | 2 |
|  | 15 | 39 | 12 | 7 | 2 | 1 |
|  | 16 | 48 | 6 | 3 | 1 | 1 |
|  | **Pot total** | **322** | **74** | **68** | **15** | **20** |
|  | 17 | 46 | 6 | 7 | 0 | 1 |
|  | 18 | 35 | 10 | 10 | 0 | 1 |
|  | 19 | 39 | 12 | 12 | 1 | 2 |
| Sample pot 3 | 20 | 21 | 15 | 10 | 2 | 3 |
|  | 21 | 37 | 8 | 3 | 5 | 0 |
|  | 22 | 22 | 3 | 13 | 0 | 2 |
|  | 23 | 44 | 22 | 9 | 0 | 1 |
|  | 24 | 21 | 8 | 4 | 2 | 1 |
|  | **Pot total** | **265** | **84** | **68** | **10** | **11** |
|  | 25 | 64 | 14 | 1 | 3 | 0 |
|  | 26 | 39 | 3 | 3 | 1 | 1 |
|  | 27 | 44 | 8 | 1 | 2 | 1 |
| Sample pot 4 | 28 | 62 | 3 | 7 | 2 | 3 |
|  | 29 | 56 | 8 | 2 | 6 | 4 |
|  | 30 | 33 | 4 | 3 | 3 | 0 |
|  | 31 | 25 | 10 | 8 | 4 | 0 |
|  | 32 | 27 | 4 | 3 | 3 | 0 |
|  | **Pot total** | **350** | **54** | **28** | **24** | **9** |
| **Total for 4 pots** | | **1129** | **299** | **200** | **69** | **49** |
| **Extrapolation to experiment total (16 pots)** | | **4516** | **1196** | **800** | **276** | **156** |
