# Supplementary material for: Mutagenesis of Puccinia graminis f. sp. tritici and Selection of Gain-of-Virulence Mutants
Source: Front Plant Sci. 2020 Sep 16;11:570180. doi: 10.3389/fpls.2020.570180 (PMC7533539; doi:10.3389/fpls.2020.570180)
Supplement: Supplementary file 7 [file Table_6.docx]

Supplementary Material

**Table S6**⎟ Correlation analysis of high confidence EMS single nucleotide variant (SNV) counts with increasing EMS concentration**.**

| **Dependent Variable: High confidence EMS SNV count** | | | | | |
| --- | --- | --- | --- | --- | --- |
| **Source** | **Type III Sum of Squares** | **df** | **MS** | **F** | **Significance** |
| **Corrected Model** | 697341.5a | 4 | 174335.375 | 16.888 | 0.021 |
| **Intercept** | 1,820,885.786 | 1 | 1820885.786 | 176.394 | 0.001 |
| **EMS Level** | 69,7341.5 | 4 | 174335.375 | 16.888 | 0.021 |
| **Error** | 3,0968.5 | 3 | 10322.833 |  |  |
| **Total** | 3,391,742 | 8 |  |  |  |
| **Corrected Total** | 728,310 | 7 |  |  |  |
| **aR Squared = 0.957 (Adjusted R Squared = 0.901)** | | | | | |
