# Supplementary material for: Mutagenesis of Puccinia graminis f. sp. tritici and Selection of Gain-of-Virulence Mutants
Source: Front Plant Sci. 2020 Sep 16;11:570180. doi: 10.3389/fpls.2020.570180 (PMC7533539; doi:10.3389/fpls.2020.570180)
Supplement: Supplementary file 8 [file Table_7.docx]

Supplementary Material

**TABLE S7**⎟ Infection types of *Pgt* UK-01 mutants virulent on *Sr43*, *Sr44*, or *Sr45.*

| **Identifier** | ***S43* IT** | **Identifier** | ***Sr44* IT** | **Identifier** | ***Sr45* IT** |
| --- | --- | --- | --- | --- | --- |
| Wildtype | 1+ | Wildtype | ; | Wildtype | 1 |
| AvrSr43 M-1 | 3 | AvrSr44 M-1 | 1+ | AvrSr45 M-1 | 3 |
| AvrSr43 M-3 | 4 | AvrSr44 M-2 | 1 | AvrSr45 M-2 | 3 |
| AvrSr43 M-4 | 3+ | AvrSr44 M-3 | 1+ | AvrSr45 M-3 | 2+ |
| AvrSr43 M-5 | 3 | AvrSr44 M-4 | 1+ | AvrSr45 M-4 | 4 |
| AvrSr43 M-6 | 4 |  |  | AvrSr45 M-5 | 4 |
| AvrSr43 M-7 | 3 |  |  | AvrSr45 M-6 | 2+ |
| AvrSr43 M-8 | 4 |  |  | AvrSr45 M-7 | 3 |
| AvrSr43 M-9 | 4 |  |  | AvrSr45 M-8 | 3 |
| AvrSr43 M-10 | 3 |  |  | AvrSr45 M-9 | 4 |
| AvrSr43 M-11 | 4 |  |  | AvrSr45 M-10 | 2+ |
| AvrSr43 M-12 |  |  |  | AvrSr45 M-11 | 2+ |
| AvrSr43 M-13 |  |  |  | AvrSr45 M-12 | 3 |
| AvrSr43 M-14 |  |  |  | AvrSr45 M-13 | 3 |
|  |  |  |  | AvrSr45 M-14 | 3 |
