# Supplementary material for: Mutagenesis of Puccinia graminis f. sp. tritici and Selection of Gain-of-Virulence Mutants
Source: Front Plant Sci. 2020 Sep 16;11:570180. doi: 10.3389/fpls.2020.570180 (PMC7533539; doi:10.3389/fpls.2020.570180)
Supplement: Supplementary file 9 [file Table_8.docx]

Supplementary Material

**TABLE S8**⎟ Race profile of eight randomly selected mutants virulent on *Sr43.*

| **Set** | **Cultivar** | **Gene** | **UK-01** | **M-1** | **M-3** | **M-4** | **M-5** | **M-6** | **M-7** | **M-8** | **M-9** | **Race** |
| --- | --- | --- | --- | --- | --- | --- | --- | --- | --- | --- | --- | --- |
| **1** |  |  | Infection type High ( > 2+) or Low (<2+) | | | | | | | | |  |
|  | ISr5-Ra CI 14159 | *Sr5* | H | H | H | H | H | H | H | H | H | **T** |
|  | *T monococcum/8*LMPG-6 DK13* | *Sr21* | H | H | H | H | H | H | H | H | H |  |
|  | Vernstein PI 442914 | *Sr9e* | H | H | H | H | H | H | H | H | H |  |
|  | ISr7b-Ra CI 14165 | *Sr7b* | H | H | H | H | H | H | H | H | H |  |
| **2** | Yalta PI 155433 | *Sr11* | L | L | L | L | L | L | L | L | L | **K** |
|  | ISr6-Ra CI 14163 | *Sr6* | H | H | H | H | H | H | H | H | H |  |
|  | Mentana W1124 PI 221154 | *Sr8a* | H | H | H | H | H | H | H | H | H |  |
|  | Acme CI 5284 | *Sr9g* | H | H | H | H | H | H | H | H | H |  |
| **3** | W2691SrTt-1 CI 17385 | *Sr36* | H | H | H | H | H | H | H | H | H | **T** |
|  | Prelude*4/2/Marquis*6/Kenya 117A | *Sr9b* | H | H | H | H | H | H | H | H | H |  |
|  | Festiguay W2706 PI 330957 | *Sr30* | H | H | H | H | H | H | H | H | H |  |
|  | Prelude/8*Marquis*2/2/Esp 518/9 | *Sr17* | H | H | H | H | H | H | H | H | H |  |
| **4** | ISr9a-Ra CI 14169 | *Sr9a* | H | H | H | H | H | H | H | H | H | **T** |
|  | ISr9d-Ra CI 14177 | *Sr9d* | H | H | H | H | H | H | H | H | H |  |
|  | W2691Sr10 CI 17388 | *Sr10* | H | H | H | H | H | H | H | H | H |  |
|  | CnsSrTmp | *SrTmp* | H | H | H | H | H | H | H | H | H |  |
| **5** | LcSr24Ag | *Sr24* | L | L | L | L | L | L | L | L | L | **F** |
|  | Kavkaz/Federation4 | *Sr31* | L | L | L | L | L | L | L | L | L |  |
|  | VPM1 | *Sr38* | H | H | H | H | H | H | H | H | H |  |
|  | McNair 701 (CI 15288) | *SrMcN* | H | H | H | H | H | H | H | H | H |  |
